# Case Report: Cetuximab in Combination With Chemotherapy for the Treatment of Multifocal Hepatic Metastases From Colorectal Cancer Guided by Genetic Tests

**DOI:** 10.3389/fonc.2021.612171

**Published:** 2021-04-06

**Authors:** Chunhui Qiu, Sidong Xie, Na Cheng, Qu Lin, Guanzhu Shen, Zhanwang Xiang, Tanxiao Huang, Xiaoni Zhang, Jingxian Duan, Li Wei, Zongheng Zheng

**Affiliations:** ^1^ Department of Hepatic Surgery, The Third Affiliated Hospital of Sun Yat-sen University, Guangzhou, China; ^2^ Department of Radiology, The Third Affiliated Hospital of Sun Yat-sen University, Guangzhou, China; ^3^ Department of Pathology, The Third Affiliated Hospital of Sun Yat-sen University, Guangzhou, China; ^4^ Department of Medical Oncology, The Third Affiliated Hospital of Sun Yat-sen University, Guangzhou, China; ^5^ Department of Radiation Oncology, The Third Affiliated Hospital of Sun Yat-sen University, Guangzhou, China; ^6^ Department of Intenational Radiology, The Third Affiliated Hospital of Sun Yat-sen University, Guangzhou, China; ^7^ Department of Oncology, HaploX Biotechnology, Shenzhen, China; ^8^ Department of Gastrointestinal Surgery, The Third Affiliated Hospital of Sun Yat-sen University, Guangzhou, China

**Keywords:** colorectal cancer, hepatic metastases, multifocal tumors, Wnt signal pathway, APC and TP53 co-mutation

## Abstract

Hepatic metastases were reported in up to 70% of colorectal cancer patients, among which multifocal hepatic metastasis represents one of the complications that lead to poor prognosis. The majority of the patients carrying multifocal hepatic metastases required pharmaceutical treatments to reduce the tumor size prior to surgical resection. However, the clinical responses to pharmaceutical agents were difficult to predict due to the heterogeneous nature of the multifocal tumors. Here, we report a case with multifocal hepatic metastases from colorectal cancer that was resistant to the primary chemotherapy and Bevacizumab plus chemotherapy, but responded to the combined therapy of Cetuximab and FOLFOX. Genetic tests had revealed that the tumor was highly metastatic due to the mutations of the WNT signaling pathway, and the metastatic tumors might be sensitive to Cetuximab. Consistent with the molecular characterizations, the metastatic tumors continue to emerge after chemotherapy, and rapidly relapsed in great numbers after liver resection. However, the combined therapy of Cetuximab and FOLFOX guided by the genetic tests significantly reduced the size and number of metastatic tumors. To conclude, deciphering the mutation profiles of multifocal metastatic tumors may guide the determination of treatment tactics, which may benefit the patients with non-resectable advanced carcinoma.

## Background

Colorectal cancer (CRC) represents the third most common cancer worldwide, causing over 500,000 deaths each year (1). Metastasis remains the key factor that shortens the survival of CRC patients. The 5-year survival of metastatic CRC was 35%-40% despite the fact that the diagnosis methods, treatment strategy, and best supportive care approaches had tremendously improved in the past decades (2). Liver remains the most common site of CRC metastasis, approximately 50% of the CRC patients developed hepatic metastasis during the course of CRC (3). Hepatic resection was considered to be the standard treatment modality for hepatic metastases from CRC (4); however, only around 25% of the patients carrying hepatic metastases from CRC were eligible for hepatic resections considering the tumor size, location, and accessibility (5, 6). To extend the possibility of hepatic resection, neoadjuvant therapy was encouraged. Neoadjuvant chemotherapy was shown to significantly improve the 5-year survival of metastatic CRC patients to 38.9%-74% (7). The combination of chemotherapy and pharmaceutical agents achieved the objective response rate of 40.6%, converting 19/64 unresectable liver metastases from colorectal cancer to curative intent resection (8). Importantly, the resistance to certain pharmaceutical agents could be postulated based on the mutational states of gene markers.

The mutational landscape of CRC was fully revealed by whole-genome sequencing studies. Mutations in APC, KRAS, BRCA2, and TP53 were frequently observed in CRC and colorectal adenoma patients (9). In hepatic metastases of CRC, KRAS was reported to be a significantly mutated gene, and the mutation status of KRAS was believed to be the predictor of recurrence-free survival of the patients (10). Patients carrying KRAS mutation had significantly decreased progression-free survival following Cetuximab treatment (11). However, KRAS mutation was detected in around 27% of colorectal liver metastases (12), and not all KRAS wild-type patients would respond to Cetuximab (13). Therefore, it is crucial to investigate the predictive biomarkers for different treatment strategies of colorectal liver metastases. This case stands for a unique representation of rapid growing multifocal hepatic metastases that were resistant to chemotherapy and Bevacizumab plus chemotherapy, and relapsed quickly after liver resection with 18 metastatic tumors. Treatment guided by the mutational profiles of the tumors effectively reduced metastases and tumor growth.

## Case Presentation

**Figure 1:**
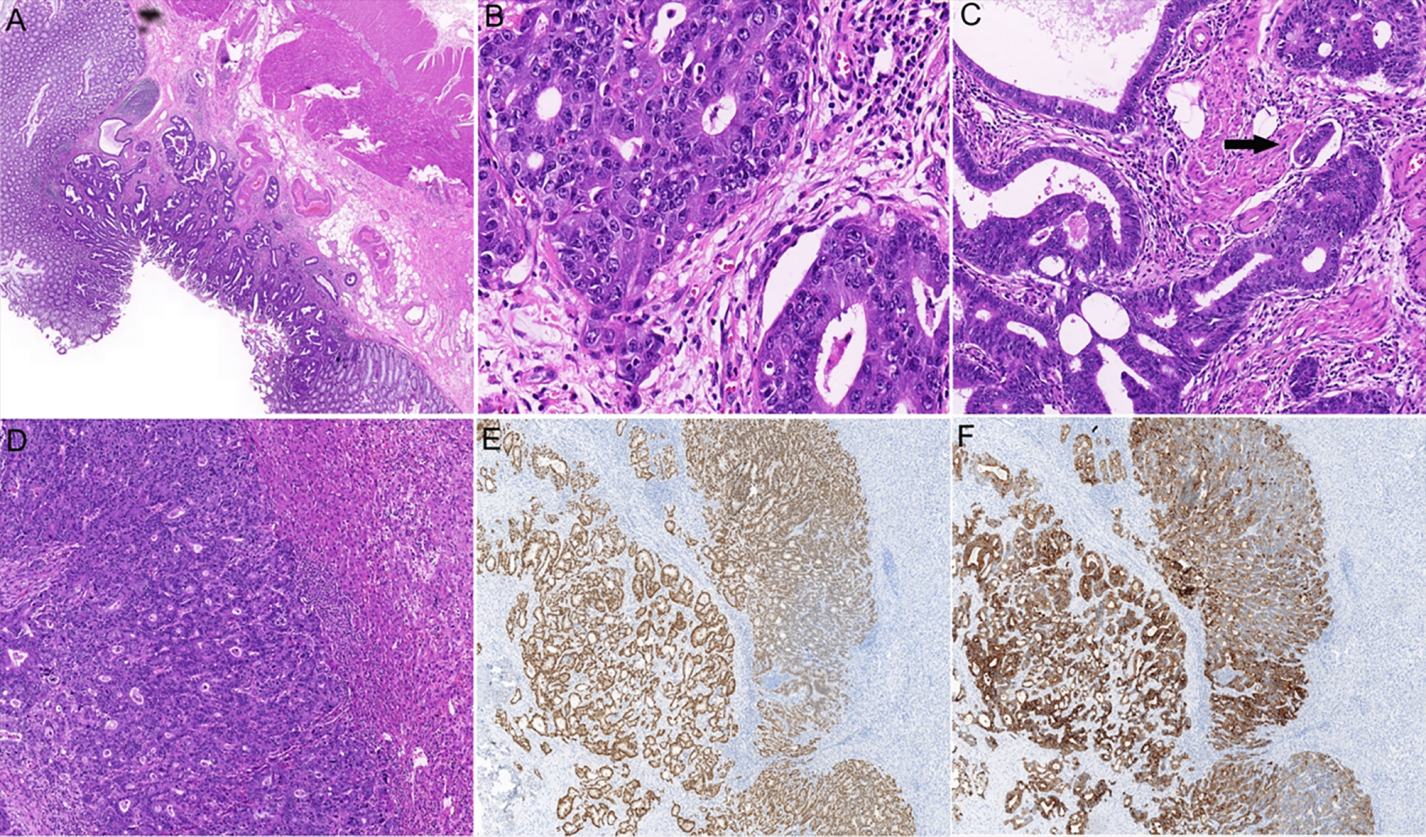
Histopathological examination of the primary and metastatic tumors. **(A)** Hematoxylin and eosin (H&E) staining of colonic primary tumor shows glandular differentiation and invasion into the submucosa (×20). **(B)** High-power view shows the malignant epithelial cells arranging in a glandular or cribriform manner (×400). **(C)** The lymphovascular invasion of colonic adenocarcinoma is indicated by an arrow (×200). **(D)** H&E staining shows intrahepatic adenocarcinoma (×100). Immunostaining for CK20 **(E)**, ×50 and CDX2 **(F)**, ×50 confirmed the tumor originated in colon.

A 51-year-old male with no family history or chronic disease was admitted to the Third affiliated hospital of Sun Yat-sen University. Baseline examination showed that carcinoembryonic antigen (CEA) was 285.6 ug/L (**Supplementary Figure 1A**), and CA199 was within the normal range on Oct 30th, 2019. Colonoscopy showed a 1.3cm x 1.3cm disc-shaped bulging tumor in the splenic flexure of colon. Pathological examination, CT scan, MR scan, and PET-CT scan showed 1 primary tumor in the splenic flexure of colon (**Figures 1A–C**) and 9 liver metastases (**Figures 1D–F**, **Supplementary Table 1** and **Supplementary Figure 2**). 3 liver metastatic tumors were identified in the left lobe of the liver, whereas the other 6 tumors were in the right lobe. The mean size of the metastatic tumors was 13 ± 2.055mm, ranging from 4mm-22mm (**Supplementary Table 1** and **Supplementary Figure 1B**). Pathological examination showed moderately differentiated adenocarcinoma of the colon (**Figures 1A–C**) and liver metastasis (**Figures 1D–F**). Targeted sequencing showed that the tumors cells were KRAS, NRAS, and BRAF wild type with proficient MMR, and was negative for HER-2.

**Figure 2:**
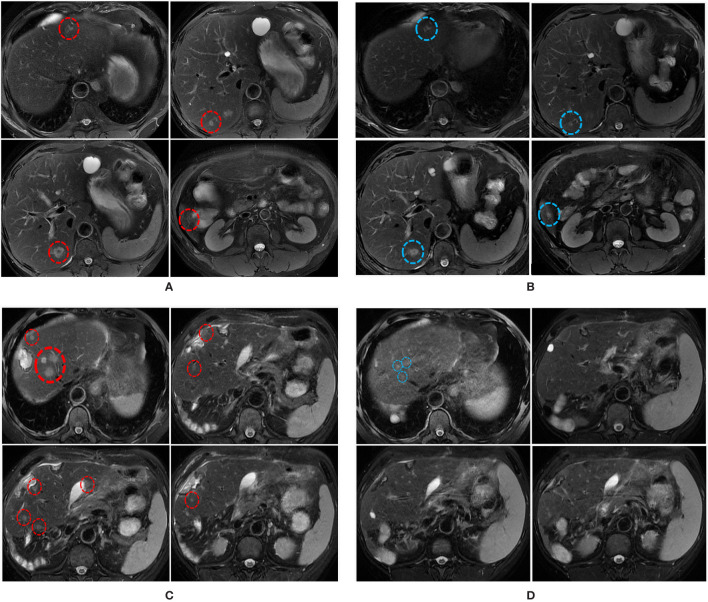
MRI scans illustrating the changes in liver metastatic tumors over time. **(A)** MRI scans on the 30^th^ of December 2019 shows multiple liver metastases (red circles). **(B)** MRI scans on the 7^th^ of March 2020 shows the size of some liver metastases was reduced (blue circles) following the combination treatment. **(C)** MRI scans on the 16^th^ of April 2020 shows multiple liver metastases (red circles). **(D)** MRI scans on the 28^th^ of June 2020 shows the sizes and numbers of liver metastasis were significantly reduced following the combined treatment.

**Figure 3:**
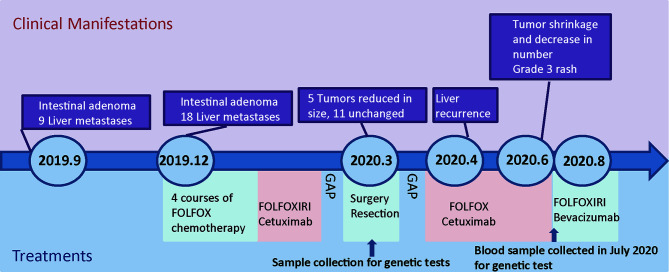
Schematic diagram showing treatment record of the patient.

From the 30^th^ of October to the 31^st^ of December, 2019, 4 courses of mFolfox6 chemotherapy were performed. The dosage and durations were Oxaliplatin 85 mg/m2 IV day 1, Leucovorin 400 mg/m2 IV day 1, 5-FU 400 mg/m2 IV bolus on day 1, then 1200 mg/m2/day x 2 days (total 2400 mg/m2 over 46–48 hours) IV continuous infusion, repeated every 2 weeks. After chemotherapy, the sizes of 7/9 tumors were reduced, the average reduction was 2.778 ± 0.7027 mm (**Figure 2A**). However, the number of the liver metastasis increased, 10 more metastatic tumors emerged in segments II-VIII, and their sizes ranged from 4mm-12mm (5.82 mm on average,**Supplementary Table 1**, **Supplementary Figures 1B**, **2**). The changes in tumor sizes and numbers after different treatments are shown in **Figures 2A–D**, and the treatment records are shown in **Figure 3**. The CEA dropped to 105 ug/L, whereas CA199 remained normal (**Supplementary Figure 1A**). To further reduce the size and number of the tumors, enhanced treatment was recommended, and the combined treatment of EGFR inhibitor and chemotherapy was applied due to the fact that the patient harbored no RAS alteration according to the mutation profile of the patient.

Two cycles of Cetuximab (500 mg/m2 IV over 2 hours, day 1, every 2 weeks) in combination with FOLFOXIRI (Irinotecan 165 mg/m2 IV day 1, oxaliplatin 85 mg/m2 IV day 1,z Leucovorin 400aa mg/m2 day 1, fluorouracil 1200 mg/m2/day x 2 days, continuous infusion starting on day 1. Repeated every 2 weeks) were administered on January 3 and 17, 2020 respectively. The patient did not return to the hospital for treatment in February due to the COVID-19 epidemic. He returned to our hospital in March 2020, his serum CEA dropped to 38.96 ug/L (**Supplementary Figure 1A**), and the CA199 remained normal. MRI scan on March 7^th^ 2020 showed that 5 out of 18 liver metastatic tumors were reduced by 3 ± 0.55 mm (**Figure 2B**, **Supplementary Table 1** and **Supplementary Figure 2**), 11 tumors remained unchanged, and two tumors enlarged by 1mm. The average size of the metastatic tumors was 7.278 ± 0.885 mm, ranging from 4mm to 17 mm.

After MDT discussion, he received surgery on the 13^th^ of March 2020. The intestinal lesion and all eighteen liver lesions were removed. Postoperative pathology examination reported middle grade adenocarcinoma of the colon with infiltration into the submucosa. Suspected vascular tumor thrombosis was also observed. T1-T7,T8-T10, T12, T13, T15-18 were identified as the metastases from the intestinal adenocarcinoma based on the immunohistochemistry staining results of CK20 and CDX2 (**Figures 1E, F**). T 11 and T14 No spare tissues were left in T11 and T14 for CK20 and CDX2 staining after DNA extraction, the H&E staining before DNA extraction confirmed that they were tumors. No metastatic cancer was found in 8 para-intestinal lymph nodes, 11 central lymph nodes, and 8 intermediate lymph nodes.

Whole-exome sequencing (WES) was performed on the intestinal adenocarcinoma sample collected during the surgery, and targeted sequencing with a panel of 680 cancer-related genes was performed on 13 hepatic tumors (T1-T7, T9-T12, T14, T15) that yielded sufficient amounts of tissues for the tests. The other 5 tumor samples did not reach the required tissue weight or tumor purity for DNA extraction, thus did not yield enough DNA for sequencing. The WES result revealed 18 somatic single-nucleotide variations and 3 insertion/deletion events (**Supplementary Table 2**). The top 5 genes with high variant allele fractions were LRRC14 (c.979C>T, 15.52%), P2RY10 (c.396del, 10.06%), GALNT2 (c.83G>A, 9.23%), CASS4 (c.719C>T, 9.18%), and PRAMEF1 (c.986A>T, 8.48%). Among the 21 mutation loci, FEM1C (c.983G>A, COSM6663046), UTRN (c.9299C>T, COSM6819331), LRRC14 (c.979C>T, COSM6703564), TP53 (c.733G>T, COSM11081), and ABCA7 (c.4513C>T, COSM2156640) were reported in the COSMIC (catalogue of somatic mutations in cancer) database; whereas the other sites of mutations were not documented in the database. The TMB of the sample was 0.53 Muts/Mb.

**Figure 4:**
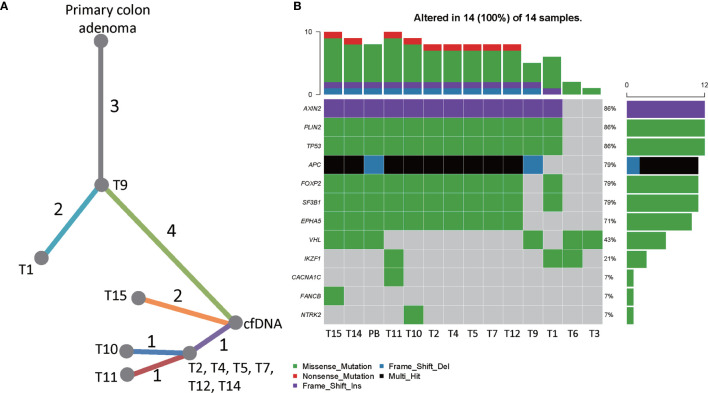
The genetic heterogeneity of liver metastatic tumors. **(A)** A phylogenic tree showing the genomic similarity of the liver metastatic tumors and the primary intestinal adenoma. **(B)** Heatmap showing the frequencies and types of mutations of all mutated genes detected by targeted sequencing. T1-T15 were hepatic tumors and PB was the peripheral blood sample obtained in July 2020.

The targeted sequencing results of the hepatic tumors revealed substantial heterogeneity among these metastatic tumors. 13 mutation loci were reported, including FOXP2 c.A455T, IKZF1 c.G1143T, TP53 c.G733T, PLIN2 c.C1295T, EPHA5 c.A2303C, APC c.C847T and c.3927_3931del, SF3B1 c.G3890A, NTRK2 c.C1574G, CACNA1C c.G673A, FANCB c.G901C, VHL c.G430A, and AXIN2 c.17_20dup. Notably, only the mutations in TP53, PLIN2, and AXIN2 were shared by the hepatic metastases and the primary intestinal adenoma. As shown in the phylogenic tree of the tumors (**Figure 4A**), T9 was the metastatic tumor that is most closely related to the primary intestinal adenoma evolutionally, they displayed common mutational sites. T1 developed two more mutation sites FOXP2 and IKF1, which was followed by T2, T4, T5, T7, T12, and T14 who further developed 4 mutations. These tumors were located at the same branch of the phylogenic tree due to the fact that they exhibited the identical mutation profile, suggesting that they may be monoclonal and perhaps emerged at the same stage (**Figure 4B**). T10, T11 and T15 were evolutionally very close to T2, T4, T5, T7, T12, and T14, they further developed one or two mutations respectively. Co-mutation of TP53 and APC occurred in T2, T4 T5, T7, T10, T11, T12, T14, and T15, suggesting that these hepatic metastases may be sensitive to Cetuximab treatment (14). Further analysis showed that these tumor cells were KRAS wild-type and pMMR. The TMB was 3.76 ± 0.86Muts/Mb, indicating that the patient may not respond well to immunotherapy. Notably, the tumors harbored TP53 and APC co-mutation had the mean TMB of 5.43 ± 0.69Muts/Mb, whereas all 4 tumors that did not contain the co-mutation had the TMB below the detection threshold.

The patient received no treatment after the surgery; however, the tumors relapsed in two months. The MRI review in April showed 18 metastases in the left lobe of the liver (segments II-VI), which possibly were recurrent tumors (**Figure 2C**). The average size of the tumors was 8.778 ± 0.712 mm, ranging from 4mm to 15mm (**Supplementary Table 3** and **Supplementary Figure 3**). The recurrent tumors were found in the liver but not the colon, suggesting that the liver metastasis harbored molecular alterations that facilitate rapid tumor growth. The sequencing results showed that key genes involved in the Wnt signaling had mutated, which favors transcription of epithelial-mesenchymal transition (EMT) supported metastasis. Although Cetuximab plus chemotherapy was not the optimal recommendation for the treatment of liver metastases, we still decided to continue with this plan as the mutation status of liver tumors and former treatment history both suggest that the tumors may respond to Cetuximab plus chemotherapy. The patient received six cycles of Cetuximab (Cetuximab 500 mg/m2 IV over 2 hours, day 1, every 2 weeks) combined with mFOLFOX6 (Oxaliplatin 85 mg/m2 IV day 1z Leucovorin 400 mg/m2 IV day 1aa 5-FU 400 mg/m2 IV bolus on day 1, then 1200 mg/m2/day x 2 days, total 2400 mg/m2 over 46–48 hours, IV continuous infusion, repeated every 2 weeks). After the treatment, 10 out of 18 tumors were eliminated, the rest 8 tumors were significantly reduced in size (10.38 ± 1.133 mm versus 6 ± 0.378 mm, p=0.008 (**Figure 2D**), **Supplementary Figure 1B**). Targeted sequencing of the cfDNAs captured from the peripheral blood sample on the 10^th^ of July 2020 showed that the mutational profile of the patient remained unchanged (**Supplementary Table 2**), confirmed that the newly emerged tumors were recurrent tumors that shared the same mutations with the surgery-removed tumors. Despite the fact that the Cetuximab plus mFOLFOX6 regime gave rise to superior clinical responses as predicted by the genetic test, the patient developed a grade 3 rash after the therapy, which seriously affected the life-quality of the patient. As the result, the patient refused to continue the Cetuximab combination therapy. On August 24, 2020, the regimen was adjusted to Bevacizumab (Bevacizumab 5 mg/kg IV, day 1 Repeat every 2 weeks) combined with FOLFOXIRI (Irinotecan 180 mg/m2 IV over 30–90 minutes, day 1 Leucovorinaa 400 mg/m2 IV infusion to match duration of irinotecan infusion, day 1 5-FU 400 mg/m2 IV bolus day 1, then 1200 mg/m2/day x 2 days; total 2400 mg/m2 over 46–48 hours; continuous infusion repeated every 2 weeks). However, severe side effects in the gastrointestinal tract markedly impaired the life quality of the patient, and the CEA level increased to 494.8 ug/L. The patient chose to stop the treatment and never returned to the hospital after two courses of Bevacizumab plus FOLFOXIRI.

## Discussion

In this case of multifocal hepatic metastases from intestinal adenoma, we reported a large number of rapid growing hepatic metastases that harbored mutations of APC, AXIN2, TP53, PLIN2, and FOXP2. The sizes and numbers of the tumors were markedly reduced by Cetuximab plus chemotherapy but not chemotherapy alone or Bevacizumab plus chemotherapy. The genomic landscape of colorectal cancers had been reported by multiple studies, APC and CTNNB1 were the hallmark mutations of CRC (15, 16). Mutations of *KRAS*, *BRAF*, *PIK3CA*, *AKT1*, *RNF43*, and *SMAD4* were observed more frequently in right-sided CRC, and alterations in the WNT signaling pathway were identified in approximately 96% of CRCs (15). The genomic landscape of liver cancer showed an overlap of prevalent mutations with CRC. TERT, TP53, and CTNNB1 were the frequently mutated genes observed in HCC patients, structural variations in CDKN2A, CCND1, APC, and TERT were also reported in virus-infected HCC (17). In hepatic metastases from CRC, COX-2 was shown to over-express (18), and KRAS mutation may predict the tumor recurrence patterns in patients who had hepatic resections of CRC metastases (10). Here, we report for the first time that TP53, PLIN2, and AXIN2 were the common mutations shared by the primary intestinal adenoma and multifocal hepatic metastases. The multifocal hepatic metastases displayed genetic heterogeneity which improves the understanding of the evolutionary and chronological relationships of the metastatic tumors.

Mutations of APC, CTNNBI or AXIN1 would lead to the accumulation of beta-catenin and interfere with the Wnt signaling pathway in HCC and CRC (19). We observed two mutation sites (in 9/13 tumors) in the exons of the APC gene, and both were loss-of-function mutation. It was shown that the inactivation of APC leads to the activation of Wnt signaling (20). Similarly, we reported the frame-shift mutation of AXIN2 in 11/13 liver metastatic tumors. This mutation results in a truncated form of protein with compromised protein function. It was known that AXIN2 was a negative regulator of the Wnt signaling pathway, hence, the observed mutation of AXIN2 may lead to the abnormal activation of the Wnt signaling pathway (21). Activation of the Wnt signaling pathway was shown to trigger the EMT-related genes including SNAIL1 (22); consequently, it would facilitate cell proliferation and the potency of invasiveness (23). In this case, it is highly likely that the Wnt signaling pathway was up-regulated due to the loss-of function mutations of APC and AXIN2, which increased the invasiveness of the tumors and caused multifocal metastases as well as recurrent metastasis in the liver. When first enrolled in the hospital, the patient developed 9 more hepatic tumors in 3 months, 8/9 of the tumors exhibiting TP53 and APC co-mutation also showed higher TMB level. After the resection, another 18 metastatic tumors emerged in the liver within two months. The rapid emerging of liver metastases may also be the result of the aberrant activation of the Wnt signaling. A population-based study reported that 45.8% liver metastases from colon cancer had 1-3 liver metastatic sites (24), the highly invasive metastases with 18 sites were rare in our clinical observation.

We also observed the co-mutation of TP53 and APC in 9/13 liver metastatic tumors. Studies had reported that the co-mutation of APC and TP53 was associated with sensitivity to Cetuximab therapy in Ras-normal metastatic CRC patients (14, 25). Indeed, FOLFIX chemotherapy alone caused an increased number of liver metastases, whereas the combined treatment of Cetuximab and chemotherapy significantly reduced the size and number of liver metastatic tumors between April 2020 and June 2020, which proved that the mutation profiles of tumors could guide the determination of clinical treatment strategy. In fact, these fast-growing metastatic tumors with loss-of function mutations of the negative regulator of the Wnt signaling pathway responded only to Cetuximab plus chemotherapy. When chemotherapy was applied alone, the number of metastatic tumors increased. The CEA level of the patient increased drastically when the treatment strategy was switched to Bevacizumab plus chemotherapy, and the sizes and number of the tumors were not reduced. Moreover, the surgery was accompanied by the rapid relapse of hepatic tumors. Despite the fact that surgical resection was considered to be the potentially curative treatment for colorectal liver metastases, our case showed that metastatic tumors with potentially Wnt signaling activating mutations may relapse very soon after the surgery, and Cetuximab plus chemotherapy was the optimal strategy for the treatment of tumors that has high potential to metastasize and multiply. Conducting genetic sequencing to check for Wnt signaling-activating mutations might be a good approach to identify such tumors. Notably, we showed that the tumors harbored loss-of function mutations of the negative regulator of the Wnt signaling pathway, which may lead to the aberrant activation of the pathway. However, we do not have spare samples to conduct immune-staining or functional tests. Whether the Wnt signaling pathway was up-regulated *in situ* requires additional experiments, and we could confirm the finding if similar samples are acquired in the future.

Systemic chemotherapy was applied as neoadjuvant therapy or adjuvant therapy in the treatment of CRC. First-line chemotherapy plans for metastatic CRC include FOLFOX (fluorouracil, leucovorin, oxaliplatin), FOLFIRI (fluorouracil, leucovorin, irinotecan), XELOX (capecitabine plus oxaliplatin), and FOLFOXFIRI (fluorouracil, leucovorin, oxaliplatin, irinotecan) (26). The combined use of Cetuximab, Bevacizumab, and chemotherapy in metastatic CRC was studied for decades; it was shown that patients carrying KRAS mutation had significantly decreased progression-free survival following Cetuximab treatment (11). A more recent study reported that the addition of Cetuximab to chemotherapy conferred disadvantages of survival in patients with operable disease (27). Collectively, these observations confirmed that the use of Cetuximab should be carefully considered, and should follow the guidance of the genetic test results. Despite the fact that the combined use of Cetuximab and chemotherapy reduced the hepatic metastases in this case, the severe adverse effect forced us to change the treatment plan to Bevacizumab plus chemotherapy, which further caused adverse effect in the gastrointestinal tract.

One limitation of the case is that we struggle to find an effective treatment strategy with less treatment-related adverse events. The disadvantage of the Cetuximab plus chemotherapy plan is that it was reported to induce skin rash in 16% of the patients, whereas in the control group (Chemotherapy alone) only 1% of patients reported skin irritation (27). Alternatively, Bevacizumab was applied in combination with chemotherapy for the treatment of metastatic CRC, the treatment plan was shown to improve the progression-free survival of the patients, although grade 3 or 4 adverse events were more frequently observed too (28). It was reported that 19.7% patients receiving FOLFIRI plus Bevacizumab and 20.4% patients receiving FOLFOXIRI plus Bevacizumab experienced serious adverse events; 20.5%-50% of the patients reported neutropenia (28). Unfortunately, the patient could not endure the severe side effects caused by the treatments and did not return to the hospital since.

## Conclusion

Next generation sequencing of the multifocal liver metastases from CRC revealed the genetic heterogeneity of the metastatic tumors. The mutations causing abnormal activation of the Wnt signaling pathway would possibly increase the invasiveness of the tumors and promote metastasis, which leads to rapid recurrence of the metastatic tumors in a short period of time after liver resection. Moreover, such tumors may respond well to Cetuximab plus chemotherapy but not chemotherapy alone or Bevacizumab plus chemotherapy. Treatment strategies should be selected based on the mutational profiles of the metastatic tumors.

## Data Availability Statement

The raw data supporting the conclusions of this article will be made available by the authors, without undue reservation.

## Ethics Statement

Written informed consent was obtained from the patient for the publication of any potentially identifiable images or data included in this manuscript.

## Author Contributions

CQ, SX, and NC contributed equally to this work. All authors were involved in the drafting of the manuscript. ZZ and LW designed the clinical treatment for the patient. JD, TH, and XZ performed genetic tests and the data analysis. ZZ, SX, GS, NC, ZX, LW, QL, and CQ performed the clinical treatment for the patients. All authors contributed to the article and approved the submitted version.

## Funding

This research was funded by the Science and Technology Planning Project of Guangdong Province, China (2015A020210044, 2014A020209022) and Shenzhen science, technology and innovation commission (JSGG20180508152646606).

## Conflict of Interest

JD, TH, and XZ were employed by the company, HaploX Biotechnology.

The remaining authors declare that the research was conducted in the absence of any commercial or financial relationships that could be construed as a potential conflict of interest.
