# Supplementary material for: Case Report: Cetuximab in Combination With Chemotherapy for the Treatment of Multifocal Hepatic Metastases From Colorectal Cancer Guided by Genetic Tests
Source: Front Oncol. 2021 Apr 6;11:612171. doi: 10.3389/fonc.2021.612171 (PMC8056263; doi:10.3389/fonc.2021.612171)
Supplement: Supplementary file 1 [file DataSheet_1.pdf]

CARE Checklist of information to include when writing a case report

CARE case report guidelines

(cc) BY-NC-ND

Topic	Item	Checklist item description	Reported on Line
Title	1	The diagnosis or intervention of primary focus followed by the words "case report"	Line 1-3
Key Words	2	2 to 5 key words that identify diagnoses or interventions in this case report, including "case report"	Line 18-19
Abstract (no references)	3a	Introduction: What is unique about this case and what does it add to the scientific literature?	Line 24-27
	3b	Main symptoms and/or important clinical findings	Line 27-29
	3c	The main diagnoses, therapeutic interventions, and outcomes	Line 27-34
	3d	Conclusion—What is the main "take-away" lesson(s) from this case?	Line 34-37
Introduction	4	One or two paragraphs summarizing why this case is unique (**may include references**)	Line 38-73
Patient Information	5a	De-identified patient specific information.	Line 75,76
	5b	Primary concerns and symptoms of the patient.	Line 75-85
	5c	Medical, family, and psycho-social history including relevant genetic information	Line 75,87-89
	5d	Relevant past interventions with outcomes	N/A
Clinical Findings	6	Describe significant physical examination (PE) and important clinical findings.	Line 76-87
Timeline	7	Historical and current information from this episode of care organized as a timeline	Line 78,91,119,167,184-185
Diagnostic Assessment	8a	Diagnostic testing (such as PE, laboratory testing, imaging, surveys).	Line 78-87,95-101,112-117,169
	8b	Diagnostic challenges (such as access to testing, financial, or cultural)	N/A
	8c	Diagnosis (including other diagnoses considered)	Line 78-87
	8d	Prognosis (such as staging in oncology) where applicable	Line 95-99,114-117,126,174-178
Therapeutic Intervention	9a	Types of therapeutic intervention (such as pharmacologic, surgical, preventive, self-care)	Line 92-95,106-109,120,170-174
	9b	Administration of therapeutic intervention (such as dosage, strength, duration)	Line 92-95, 106-109, 170-174,185
	9c	Changes in therapeutic intervention (with rationale)	Line 101-104, 181-190
Follow-up and Outcomes	10a	Clinician and patient-assessed outcomes (if available)	Line 113-117,174-176
	10b	Important follow-up diagnostic and other test results	Line 174-179
	10c	Intervention adherence and tolerability (How was this assessed?)	N/A
	10d	Adverse and unanticipated events	Line 182-184
Discussion	11a	A scientific discussion of the strengths AND limitations associated with this case report	Line 318-323,234-293,
	11b	Discussion of the relevant medical literature **with references**.	Line 295-316
	11c	The scientific rationale for any conclusions (including assessment of possible causes)	Line 269-284, 286-293
	11d	The primary "take-away" lessons of this case report (without references) in a one paragraph conclusion	Line 326-331
Patient Perspective	12	The patient should share their perspective in one to two paragraphs on the treatment(s) they received	N/A
Informed Consent	13	Did the patient give informed consent? Please provide if requested	Yes ☑ No ☐
