# Supplementary material for: Case Report: Cetuximab in Combination With Chemotherapy for the Treatment of Multifocal Hepatic Metastases From Colorectal Cancer Guided by Genetic Tests
Source: Front Oncol. 2021 Apr 6;11:612171. doi: 10.3389/fonc.2021.612171 (PMC8056263; doi:10.3389/fonc.2021.612171)
Supplement: Supplementary file 2 [file DataSheet_2.pdf]

Supplementary Figures

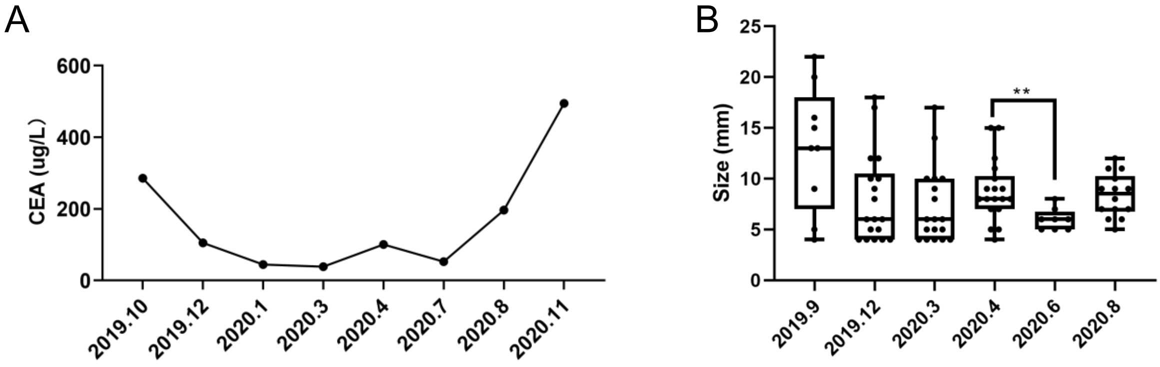

Supplementary Figure 1 Changes in CEA and tumor size over time

(A) Carcinoembryonic antigen (CEA,ug/L) level in peripheral blood. (B) Tumor sizes are (mm) presented with the largest dimension of the tumor. Data were compared by paired T test, ** shows p<0.001.

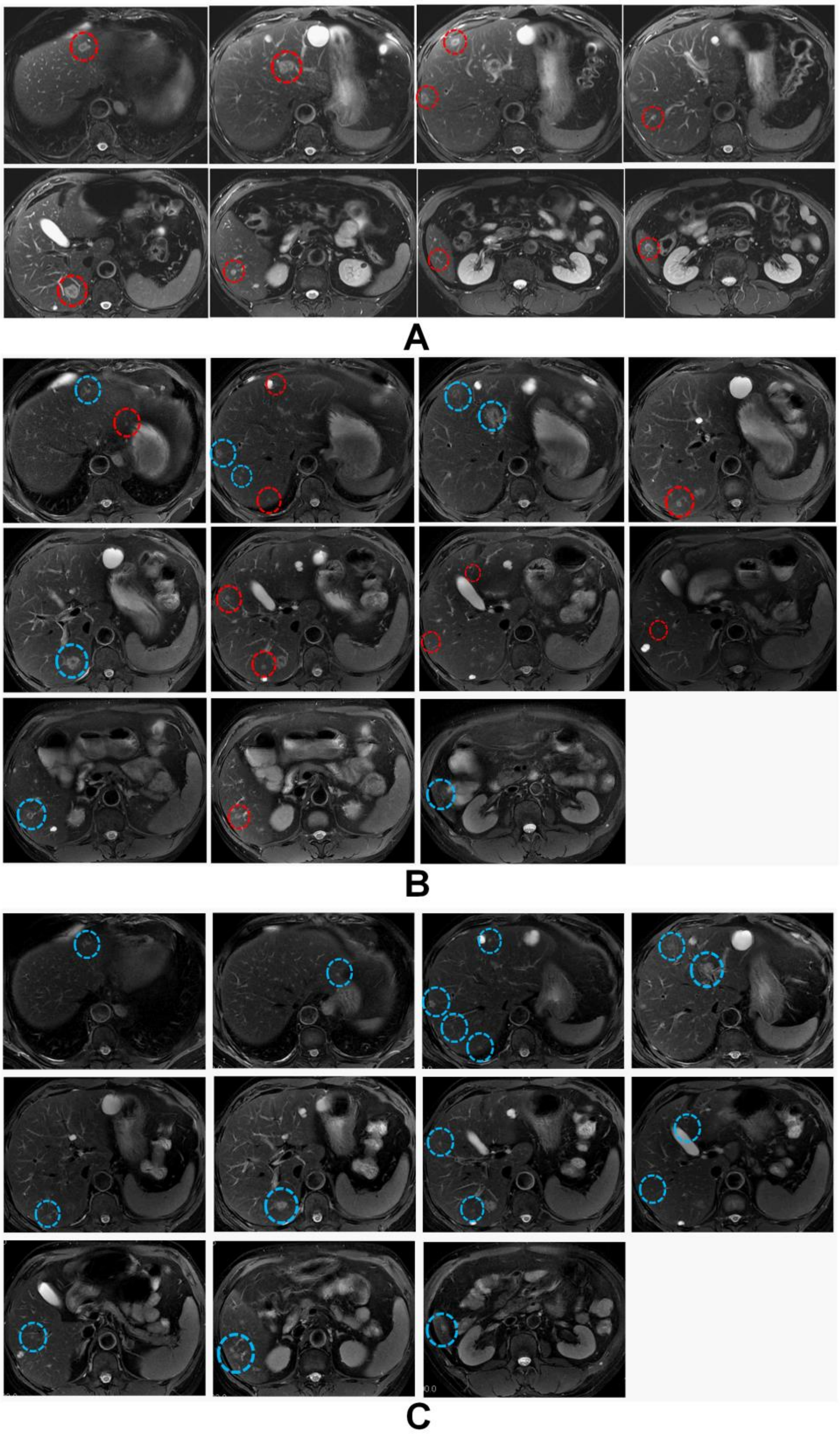

Supplementary Figure 2 MRI scans of the liver metastatic tumor before surgical resection

(A) MRI scans on the 23th of September 2019 showing multiple liver metastases. (B) MRI scans on the 30th of December 2019. (C) MRI scans on the 7th of March 2020. Red circles stands for newly emerged tumors, and blue circles labels remaining

tumors.

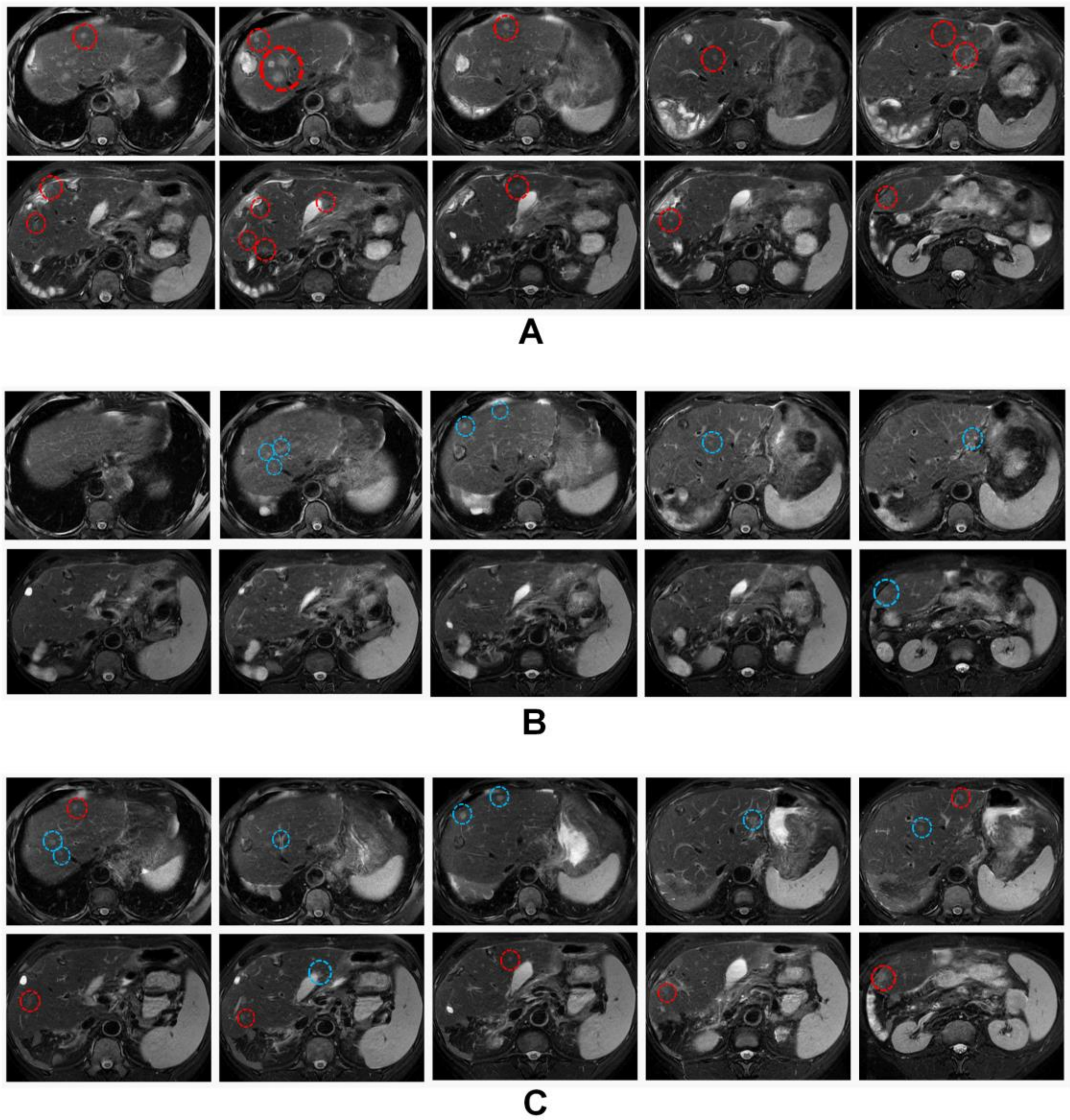

Supplementary Figure 3 MRI scans of the liver metastatic tumor after surgical resection

(B) MRI scans on the 16th of April 2020 showing multiple liver metastases. (B) MRI scans on the 28th of June 2020. (C) MRI scans on the 30th of August 2020. Red circles stands for newly emerged tumors, and blue circles labels remaining tumors.
