# Supplementary material for: Case Report: Cetuximab in Combination With Chemotherapy for the Treatment of Multifocal Hepatic Metastases From Colorectal Cancer Guided by Genetic Tests
Source: Front Oncol. 2021 Apr 6;11:612171. doi: 10.3389/fonc.2021.612171 (PMC8056263; doi:10.3389/fonc.2021.612171)
Supplement: Supplementary file 3 [file Table_1.docx]

**Supplementary Tables**

**Supplementary Table 1 Tumor sizes and locations before surgical resection**

|  | 23/09/2019 | | 30/12/2019 | | 07/03/2020 |
| --- | --- | --- | --- | --- | --- |
| Tumor | Locations | Sizes (mm) | Locations | Sizes (mm) | Sizes (mm) |
| T1 | S2/4 | 16 | S2/4 | 12 | 8 |
| T2 | S2/3/4 | 22 | S2/3/4 | 17 | 17 |
| T3 | S4 | 13 | S4 | 10 | 10 |
| T4 | S7/8 | 15 | S7/8 | 10 | 10 |
| T5 | S7 | 4 | S7 | 4 | 4 |
| T6 | S6/7 | 20 | S6/7 | 18 | 14 |
| T7 | S6 | 9 | S6 | 9 | 10 |
| T8 | S5/6 | 5 | S5/6 | Eliminated | Eliminated |
| T9 | S5/6 | 13 | S5/6 | 12 | 9 |
| T10 |  |  | S2 | 5 | 6 |
| T11 |  |  | S7 | 5 | 5 |
| T12 |  |  | S7 | 8 | 5 |
| T13 |  |  | S5/8 | 6 | 6 |
| T14 |  |  | S6/7 | 6 | 5 |
| T15 |  |  | S5/6 | 6 | 6 |
| T16 |  |  | S5/6 | 4 | 4 |
| T17 |  |  | S4 | 4 | 4 |
| T18 |  |  | S5/6 | 4 | 4 |
| T19 |  |  | S4 | 4 | 4 |


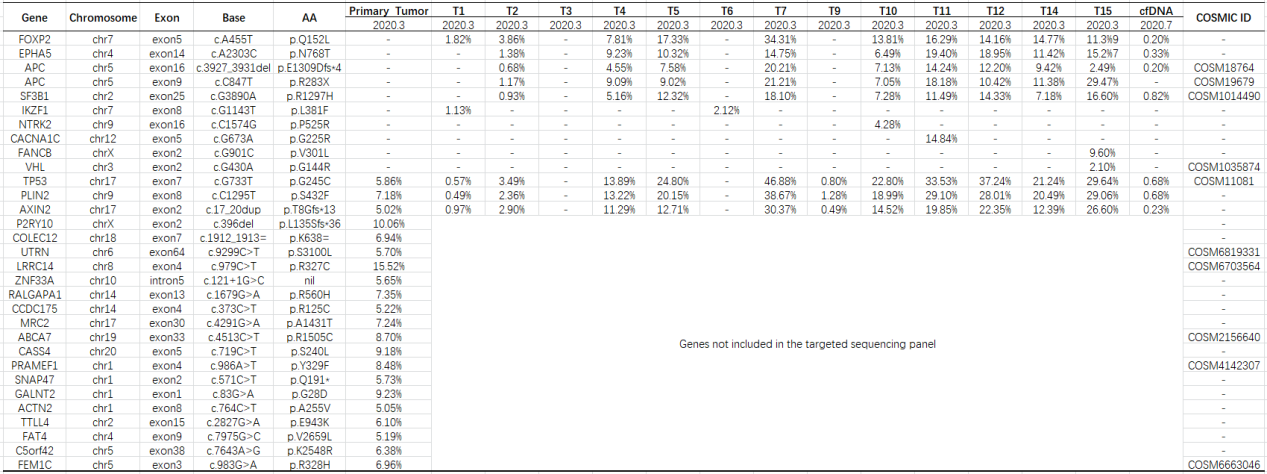


The chromosome and exon location of the mutated genes were shown, the specific mutation loci (Base) and caused protein changes (AA) were displayed. The variant allele fractions were represented as percentages.

**Supplementary Table 2 Variant allele fractions of the mutated genes detected by whole-exome sequencing and targeted sequencing**

**Supplementary Table 3 Tumor sizes and locations after surgical resection**

|  |  | 16/04/2020 | 28/06/2020 | 30/08/2020 |
| --- | --- | --- | --- | --- |
| Tumor | Locations | Sizes (mm) | Sizes (mm) | Sizes (mm) |
| T’1 | S2 | 9 | Eliminated | 7 |
| T’2 | S2 | 8 | 6 | 9 |
| T’3 | S2 | 8 | 5 | 9 |
| T’4 | S2 | 12 | 7 | 11 |
| T’5 | S2 | 5 | Eliminated | Eliminated |
| T’6 | S2 | N/A | N/A | 10 |
| T’7 | S2/4 | 15 | 6 | 10 |
| T’8 | S2/4 | 15 | 5 | 7 |
| T’9 | S2/4 | 9 | 6 | 7 |
| T’10 | S2/4 | 7 | 5 | 9 |
| T’11 | S3 | 8 | Eliminated | 8 |
| T’12 | S3 | 5 | Eliminated | 6 |
| T’13 | S4b | 11 | Eliminated | Eliminated |
| T’14 | S4b | 10 | Eliminated | Eliminated |
| T’15 | S4b | 7 | Eliminated | 11 |
| T’16 | S4b | 8 | Eliminated | 6 |
| T’17 | S4b | 8 | Eliminated | Eliminated |
| T’18 | S4b | 4 | Eliminated | 5 |
| T’19 | S4b | 9 | 8 | 12 |
